# Diabetes Attenuates the Contribution of Endogenous Nitric Oxide but Not Nitroxyl to Endothelium Dependent Relaxation of Rat Carotid Arteries

**DOI:** 10.3389/fphar.2020.585740

**Published:** 2021-01-21

**Authors:** Jasmin Chendi Li, Anida Velagic, Cheng Xue Qin, Mandy Li, Chen Huei Leo, Barbara K. Kemp-Harper, Rebecca H. Ritchie, Owen L. Woodman

**Affiliations:** ^1^ Drug, Discovery Biology, Monash Institute of Pharmaceutical Sciences, Monash University, Parkville, VIC, Australia; ^2^ Baker Heart & Diabetes Institute, Melbourne, VIC, Australia; ^3^ Department of Pharmacology, University of Melbourne, Parkville, VIC, Australia; ^4^ Department of Pharmacology, Cardiovascular Disease Program, Biomedicine Discovery Institute, Monash University, Clayton, VIC, Australia; ^5^ Science, Maths and Technology Cluster, Singapore University of Technology & Design, Singapore, Singapore

**Keywords:** nitric oxide, nitroxyl, Diabetes, endothelium, carotid arteries, nitroxyl mediated relaxation in diabetes

## Abstract

Endothelial dysfunction is a major risk factor for several of the vascular complications of diabetes, including ischemic stroke. Nitroxyl (HNO), the one electron reduced and protonated form of nitric oxide (NO•), is resistant to scavenging by superoxide, but the role of HNO in diabetes mellitus associated endothelial dysfunction in the carotid artery remains unknown.

**Aim:** To assess how diabetes affects the role of endogenous NO• and HNO in endothelium-dependent relaxation in rat isolated carotid arteries.

**Methods:** Male Sprague Dawley rats were fed a high-fat-diet (HFD) for 2 weeks prior to administration of low dose streptozotocin (STZ; 35 mg/kg i. p./day) for 2 days. The HFD was continued for a further 12 weeks. Sham rats were fed standard chow and administered with citrate vehicle. After 14 weeks total, rats were anesthetized and carotid arteries collected to assess responses to the endothelium-dependent vasodilator, acetylcholine (ACh) by myography. The combination of calcium-activated potassium channel blockers, TRAM-34 (1 μmol/L) and apamin (1 μmol/L) was used to assess the contribution of endothelium-dependent hyperpolarization to relaxation. The corresponding contribution of NOS-derived nitrogen oxide species to relaxation was assessed using the combination of the NO• synthase inhibitor, L-NAME (200 μmol/L) and the soluble guanylate cyclase inhibitor ODQ (10 μmol/L). Lastly, L-cysteine (3 mmol/L), a selective HNO scavenger, and hydroxocobalamin (HXC; 100 μmol/L), a NO• scavenger, were used to distinguish between NO• and HNO-mediated relaxation.

**Results:** At study end, diabetic rats exhibited significantly retarded body weight gain and elevated blood glucose levels compared to sham rats. The sensitivity and the maximal relaxation response to ACh was significantly impaired in carotid arteries from diabetic rats, indicating endothelial dysfunction. The vasorelaxation evoked by ACh was abolished by L-NAME plus ODQ, but not affected by the apamin plus TRAM-34 combination, indicating that NOS-derived nitrogen oxide species are the predominant endothelium-derived vasodilators in sham and diabetic rat carotid arteries. The maximum relaxation to ACh was significantly decreased by L-cysteine in both sham and diabetic rats, whereas HXC attenuated ACh-induced relaxation only in sham rats, suggesting that diabetes impaired the contribution of NO•, whereas HNO-mediated vasorelaxation remained intact.

**Conclusion:** Both NO• and HNO contribute to endothelium-dependent relaxation in carotid arteries. In diabetes, NO•-mediated relaxation is impaired, whereas HNO-mediated relaxation was preserved. The potential for preserved HNO activity under pathological conditions that are associated with oxidative stress indicates that HNO donors may represent a viable therapeutic approach to the treatment of vascular dysfunction.

## Introduction

Diabetes is a metabolic disease associated with progressive damage to the vascular wall, which can promote the development of macrovascular and microvascular complications (Stratton et al., 2006; Cade, 2008; Fowler, 2008; Fatehi-Hassanabad et al., 2010). The hallmark of these vascular complications is the development of endothelial dysfunction and increased reactive oxygen species (ROS) production (Schalkwijk, 2005; Sharma et al., 2012). This is characterized by reduced production of endothelium-derived relaxing factors including prostacyclins (Moncada et al., 1976), nitrogen oxide species such as nitric oxide (NO•) (Palmer et al., 1988) and a non-NO•/non-prostanoid mediator of endothelium-dependent hyperpolarization (EDH, previously associated with the definition of endothelium-derived hyperpolarizing factors) (Coleman et al., 2017; Garland and Dora, 2017; Leung and Vanhoutte, 2017). In addition, endothelial dysfunction is associated with atherosclerotic plaque formation, which can lead to arterial stenosis and thrombosis (Endemann and Schiffrin, 2004; Sitia et al., 2010; Zardi and Afeltra, 2010) Atherothrombotic occlusion is more prevalent in large arteries, such as carotid arteries, rather than smaller, resistance vessels. Thus, carotid artery stenosis is widely used to predict the likelihood of stroke (Dempsey et al., 2010; Kwee et al., 2013) and carotid plaque formation (Spagnoli et al., 2004; Ding et al., 2008). Given the relationship between endothelial dysfunction, atherosclerosis, and ischemic stroke, it is likely that the extent of endothelial dysfunction in the carotid vasculature may reflect the risk of an individual developing ischemic cerebrovascular disease.

There are multiple mechanisms of endothelium-dependent relaxation including via endogenous NO• (Palmer et al., 1988), HNO, the one-electron reduced and protonated form of NO• (Dutton et al., 2004; Andrews et al., 2009; Bullen et al., 2011), the arachidonic acid metabolite prostacyclin (Moncada et al., 1976) and EDH, which causing hyperpolarization in the smooth muscle layer and affecting conducted vasodilatation in arteries (Coleman et al., 2017; Garland and Dora, 2017; Leung and Vanhoutte, 2017). Importantly, like NO•, there is evidence to suggest that HNO is endogenously generated and serves as an endothelium-derived vasodilator in both conduit and resistance arteries (Ellis et al., 2000; Andrews et al., 2009; Bullen et al., 2011; Leo et al., 2012; Tare et al., 2017). While there are a range of chemical reactions that may lead to the endogenous synthesis of HNO (Marti et al., 2017), the strongest body of evidence indicates it is synthesized as a co-product of endothelial nitric oxide synthase (eNOS) during the conversion of L-arginine to NO• (Stoll et al., 2010; Paolocci et al., 2016; Marti et al., 2017). Importantly, it has also been shown that where eNOS is uncoupled due to oxidative stress or where there is a deficiency of the cofactor, tetrahydrobiopterin (BH_4_), the production of HNO by eNOS is promoted over NO• (Fukuto et al., 1992; Rusche et al., 1998; Adak et al., 2000; Tantillo et al., 2000). Thus, in disease states, where eNOS is uncoupled, HNO may be generated. While it is well established that diabetes has a detrimental effect on endothelial function which then contributes to diabetes-induced morbidity and mortality due to cardiovascular disease, it is less well established how the different mechanisms of endothelium-dependent relaxation are individually impacted. It is however clear that diabetes impairs NO• mediated relaxation associated with diabetes-induced oxidative stress. Unlike NO•, HNO is resistant to scavenging by reactive oxygen species (ROS) such as superoxide (•O_2_^−^) (Miranda et al., 2002; Leo et al., 2012), suggesting that the actions of HNO may be preserved in diabetes and HNO may be able to compensate for impaired NO•-mediated signaling. Our previous studies have shown that HNO is preserved in the diabetic aorta (Leo et al., 2012), femoral and mesenteric arteries (Kahlberg et al., 2016; Tare et al., 2017) however it is unknown if this is also the case in the carotid artery, a clinically important blood vessel given the prevalence of carotid artery stenosis and stroke in patients with diabetes.

There are contradictory findings in regard to the impact of diabetes on EDH-mediated vascular relaxation with some reports that relaxation is impaired (Kamata et al., 2000; Wigg et al., 2001; Matsumoto et al., 2003; Leo et al., 2011; Matsumoto et al., 2017) contrasted by others that EDH relaxation is maintained (Kagota et al., 2011; Cho et al., 2013; Mokhtar et al., 2016a) or even enhanced in human subcutaneous arteries (Mokhtar et al., 2016b). Although EDH does not make a significant contribution to endothelium-dependent relaxation in carotid arteries under non-disease conditions, there is evidence that it may be upregulated early in the development of diabetes (Leo et al., 2010; Centeno et al., 2019) and there is an associated increase in IK_Ca_ (Kagota et al., 2011; Schach et al., 2014). As such when considering the interplay between endothelium-derived relaxing factors in diabetes, it is important to evaluate the role of NO•, EDH and HNO.

It remains unclear whether endogenous HNO-mediated vasorelaxation and the release of basal HNO is affected by diabetes-induced endothelial dysfunction in conduit vessels such as the carotid arteries. We hypothesized that basal and stimulated release of endogenous HNO is preserved, whereas endothelial function is impaired, in diabetic rat carotid arteries. Therefore, this study aims to characterize the relative contribution of NO• and HNO to endothelium-dependent relaxation in rat carotid arteries both in terms of their basal and stimulated release.

## Materials and Methods

### Animal Model

All animal research and procedures involved in this project were conducted in accordance with the National Health and Medical Research Council of Australia Code of Practice for the Care and Use of Animals for Scientific Purposes and approved by the Alfred Medical Research Educational Precinct (AMREP) Animal Ethics Committee (AEC; under the ethics approval number: E/1759/2017/B). Male Sprague Dawley (SD) rats were bred and housed within the AMREP precinct animal center at an ambient temperature of 22°C, with a 12-h light/dark cycle. At 8 weeks of age, pre-adolescent male SD rats (n = 39; body weight: 200–350 g) were randomly allocated to one of two groups, sham or diabetic. Rats were fed an HFD (SF03–002, 36% fat and 19.4% protein with total 59% digestible energy intake from lipids, Specialty Feeds, WA, Australia) (Marsh et al., 2009) for two weeks, after which the rats were administered two low-doses of streptozotocin (STZ, 24 h apart, each 35 mg/kg i.p., in 0.1 mol/L citrate, pH4.5, n = 20). The HFD then continued for a further 12 weeks. The sham group received two injections of vehicle (24 h apart, 0.1 mol/L citrate vehicle, pH 4.5) and were fed standard laboratory chow (n = 19). Throughout the 14-weeks study period, blood samples were collected fortnightly through a small cut on the tail end and blood glucose levels were assayed by a one-touch glucometer (Roche, Sydney, NSW, Australia). The upper limit of detection of the glucometer was 33.3 mmol/L. Hence, any reading above this point was recorded as 33.3 mmol/L (Ritchie et al., 2012). One week following STZ administration, diabetic rats with blood glucose levels exceeding 28 mM received subcutaneous insulin (1–2 U as required, Humulin NPH, Lilly) to prevent complications of severe hyperglycemia. At 21 weeks of age, a glucose tolerance test (GTT) was performed. To perform the GTT, after a 6 h fast, the animals were injected with 3 ml/kg body weight of glucose solution (10% glucose w/v, i.p.) and blood glucose levels were measured at 0, 15, 30, 45, 60, 90 and 120 min via the tail vein. Body composition was determined using an EchoMRI 3-in-1 Body Composition Analyzer (Echo Medical Systems, Houston, TX, USA) to determine the body composition according to the manufacturer’s protocol. Blood glucose and glycated hemoglobin (HbA1_c_) levels were measured before exsanguination, using the one-touch glucometer and a Cobas HbA1c analyser (Roche, Sydney, NSW, Australia), respectively. Any reading is shown as “Low”, was recorded as 3% as the lower limit of detection of the HbA1_c_ analyser (Genc et al., 2012). Rats were then anesthetized by a combined dose of ketamine and xylidine (100 and 20 mg/kg, respectively, i.p.). Once anesthetized, whole blood was collected from the hepatic vein for further measurement of plasma insulin, and animals were euthanized by exsanguination (Hickman and Johnson, 2011). Both carotid arteries were collected for myography experiments. Plasma insulin was measured using commercially available Rat Ultrasensitive Insulin ELISA kits (ALPCO, Salem, NH, USA) (French et al., 2017) according to the manufacturer’s instructions.

### Myograph Experiments

#### Isolation and Equilibration of Rat Carotid Arteries

Carotid arteries were isolated and immediately placed in ice-cold Krebs’ solution (in mmol/L: 120 NaCl, 5 KCl, 1.2 MgSO_4_, 1.2 KH_2_PO_4_, 25 NaHCO_3_, 11.1 D-glucose and 2.5 CaCl_2_). Indomethacin (10 μmol/L), a non-selective cyclooxygenase inhibitor, was added to the Krebs’ solution to prevent the synthesis of prostanoids by the artery segments in all studies, as described previously (Leo et al., 2010; Kahlberg et al., 2016). Carotid arteries were cleared of all loose connective tissue and fat and cut into two- to 3-mm ring segments. Each ring was mounted on a myograph (model 610M, Danish Myo Technology, Aarhus, Denmark) containing Krebs’ solution gassed with carbogen (95% O_2_ and 5% CO_2_) at 37°C. After the arteries were mounted on the myograph, they were adjusted to a passive tension of 15 mN, which was continuously recorded using LabChart 8 Pro software (ADInstruments, Hastings, United Kingdom). Twenty minutes after equilibration, Krebs’ solution was replaced with a high K^+^-containing physiological saline solution (KPSS in mmol/L: KCL 125, MgSO_4_ 1.2, KH_4_PO_4_ 1, NaHCO_3_ 25, D-glucose 11.1, CaCl_2_ 2.5) for 20 min to induce maximal contraction (Supplementary Table S2). Vessels were then rinsed with Krebs’ solution and allowed to regain basal tension. Arteries were then precontracted to 50–70% of their maximal contraction to KPSS, using PE (0.01–1 μmol/L) in all studies. In a limited number of experiments, the thromboxane A_2_ receptor agonist 9, 11-dideoxy-9α, 11α-methanoepoxy-PGF_2α_ (U-46619; 1–10 nmol/L) was also added when arteries failed to reach optimal stable contraction with PE alone, as previously described (Kahlberg et al., 2016; Leo et al., 2020) (Supplementary Table S2). Endothelial integrity was determined by exposure to a single concentration of acetylcholine (ACh 10 μmol/L), with relaxation >80% of pre-constriction levels accepted as indicative of a functionally intact endothelium.

#### Assessment of Vascular Reactivity *Ex Vivo*

After further rinses and recovery of basal tension, arteries were again pre-contracted to 50–70% of the maximum contraction (KPSS response) using phenylephrine (PE; 0.01–1 μmol/L) either alone or in combination with U-46619 (1–10 nmol/L). The effects of different treatments on relaxation responses in carotid arteries were assessed via cumulative concentration-response curves to the endothelium-dependent vasodilator, ACh (0.1–10 μmol/L) and the endothelium-independent vasodilator, sodium nitroprusside (SNP; 0.1 nmol/l-10 μmol/L). Responses to ACh and SNP were also examined following 20 min incubation with different combinations of N^ω^-nitro-L-arginine methyl ester (L-NAME; 200 μmol/L, a NOS inhibitor); 1H-[1,2,4]oxadiazolo [4,3-a]quinoxalin-1-one (ODQ; 10 μmol/L, a soluble guanylate cyclase (sGC) inhibitor); apamin (1 μmol/L, small-conductance Ca^2+^-activated K^+^ channel (SK_Ca_) blocker); and 1-[(2-chlorophenyl) (diphenyl)methyl]-1H-pyrazole (TRAM-34; 1 μmol/L, a selective intermediate-conductance Ca^2+^-activated K^+^ channel (IK_Ca_) blocker). In addition, the K_Ca_ channel blockers were also incubated either alone or in combination with L-cysteine (3 mmol/L), a selective HNO scavenger, or hydroxocobalamin (HXC), a selective NO• scavenger (100 μmol/L), as described previously (Ritchie et al., 2013; Marshall et al., 2020).

#### Assessment of Basal Nitrogen Oxide, NO• and HNO Activity *Ex Vivo*

In another set of myograph experiments, the impact of diabetes on the basal release of eNOS-derived nitrogen oxides were determined. Endothelium-intact carotid artery rings were sub-maximally precontracted to ∼20% of the KPSS response with PE (10–100 nmol/L) (Supplementary Figures S2, S5). After stabilization of contraction, carotid artery rings were either exposed to L-NAME (200 μmol/L), L-cysteine (3 mmol/L) or HXC (100 μmol/L) (Leo et al., 2011; Kahlberg et al., 2016). Under these conditions, contractile response to L-NAME was considered to reflect the level of basal eNOS-derived nitrogen oxides (NO• and HNO). The contractile response to L-cysteine or HXC were considered to reflect the basal level of HNO or NO•, respectively.

### Reagents

All reagents were purchased from Sigma-Aldrich (St Louis, MO, USA) except for ODQ and U46619 (Cayman Chemical, Ann Arbor, MI, USA), and all compounds used were of analytical grade or higher. Aliquots of drugs were dissolved in distilled water and stored at −20°C, except indomethacin, which was dissolved in 0.1 mol/L sodium bicarbonate, as well as both ODQ and TRAM-34, which were dissolved in 100% dimethyl sulfoxide (DMSO, final concentration less than 0.1%), and U-46619, which was dissolved in absolute ethanol as a 1 mM stock solution, with subsequent dilutions in distilled water.

### Statistical Analysis

All data are expressed as mean ± SEM, where *n* is the number of animals per group. Individual concentration-response curves from rat isolated carotid arteries were computer-fitted to a sigmoidal logistical equation using non-linear regression (GraphPad Prism 7.0 Software, CA, USA) to calculate the^–^log_10_ of the concentration of each agonist causing a 50% relaxation (pEC_50_; mol/L). Maximum relaxation (R_max_) evoked by ACh and SNP were expressed as a percentage reversal of the precontraction to phenylephrine and/or U46619. Group pEC_50_, R_max_ and systemic characteristics were compared using Student’s *t*-test or one-way ANOVA followed by a *post-hoc* Dunnett’s test as appropriate. Body weights and blood glucose levels were analyzed using a two-way ANOVA with Sidak’s *post-hoc* analysis for multiple comparisons. *p* values of <0.05 were considered statistically significant.

## Results

### Systemic Characteristics *In Vivo*

**FIGURE 1:**
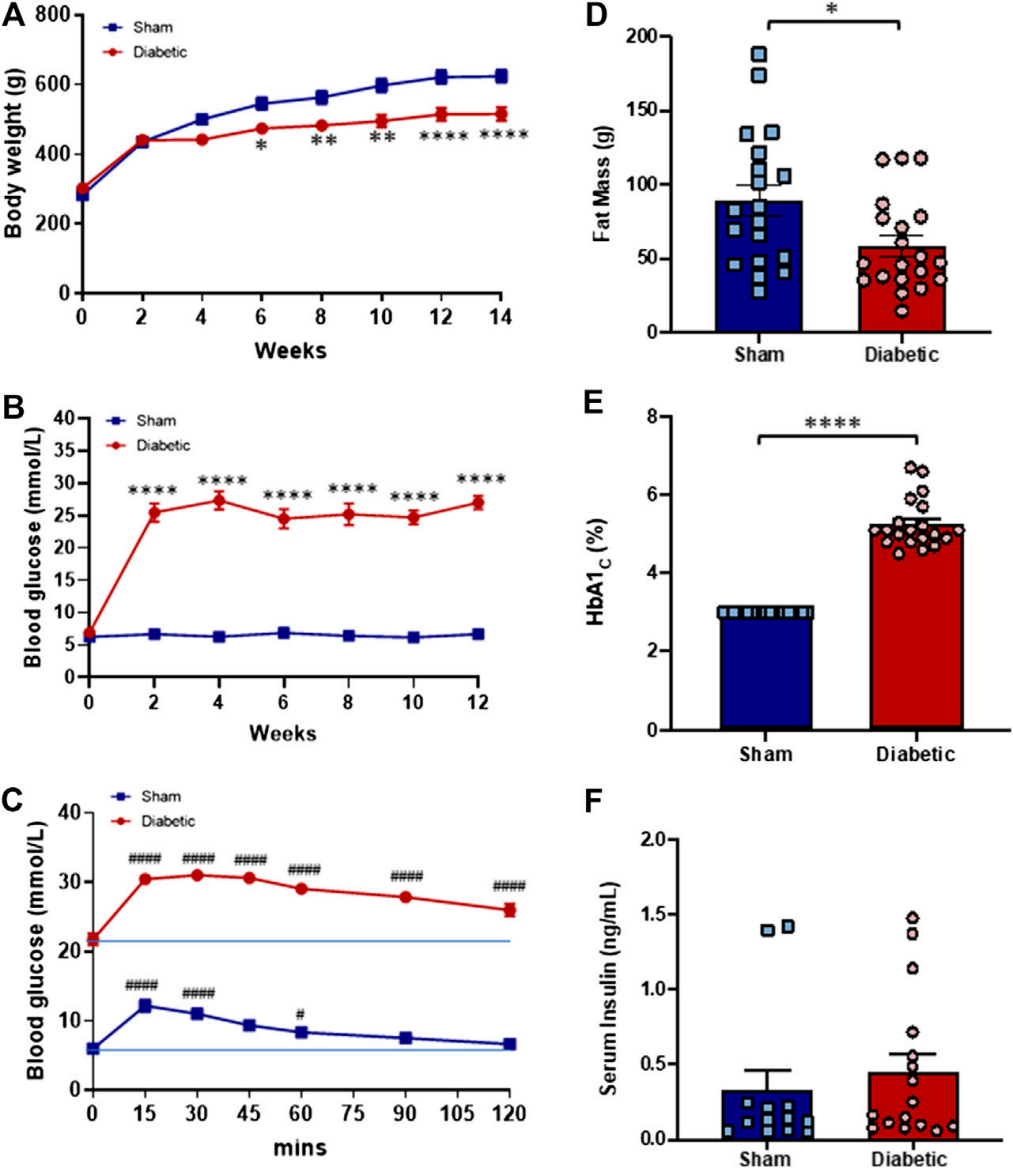
Systemic characteristics *in vivo* **.** Body weight **(A)** and blood glucose level **(B)** of sham (■) and diabetic (●) rats thought out the study period. The Blood glucose level of sham (■) and diabetic (●) rats throughout the glucose tolerance test **(C)**. Fat mass **(D)**, % HbA1c **(E)** and serum insulin level **(F)** of sham (blue bar) and diabetic (red bar) groups at the end of the experimental period. Body weight and blood glucose level (Two-way ANOVA with Sidak’s multiple comparisons test); Fat mass, % HbA_1c_ and serum insulin level (Unpaired Student’s *t*-test); Glucose tolerance test (Two-way ANOVA with Tukey’s multiple comparisons test). Diabetic n = 20; sham n = 19 experiments. Values are mean ± SEM *p < 0.05, **p < 0.01, ****p < 0.0001 vs sham animals. #*p* < 0.05, ####*p* < 0.0001 vs. zero time point.

Twelve weeks after STZ or vehicle administration and fourteen weeks after commencing HFD or standard laboratory diet, weight gain was evident in both groups, however, diabetic rats exhibited significantly lower body weight (Figure 1A) and fat mass (Figure 1D) than sham rats at end point, indicative of retarded body weight gain. Both blood glucose levels and HbA1c of diabetic rats were significantly greater than those of sham rats (Figure 1B,E).

### Glucose Tolerance Test and Plasma Insulin Level

To determine the level of glucose tolerance a GTT was performed during the final week of the study. Blood glucose levels of both sham and diabetic rats increased following an i. p. injection of glucose (3 ml/kg, 10% glucose w/v) (Figure 1C). In sham rats, the maximal rise in blood glucose concentration was evident 15 min post-injection but returned to baseline within 90 min of glucose administration. In contrast, blood glucose concentration remained elevated in diabetic rats throughout the test and did not return to baseline at test completion (120 min post-injection). These findings suggest that glucose tolerance is impaired in the diabetic group. Serum insulin concentrations measured at study endpoint did not differ significantly between the two groups (Figure 1F).

### Effect of Diabetes on Relaxation to Acetylcholine and Sodium Nitroprusside

**FIGURE 2:**
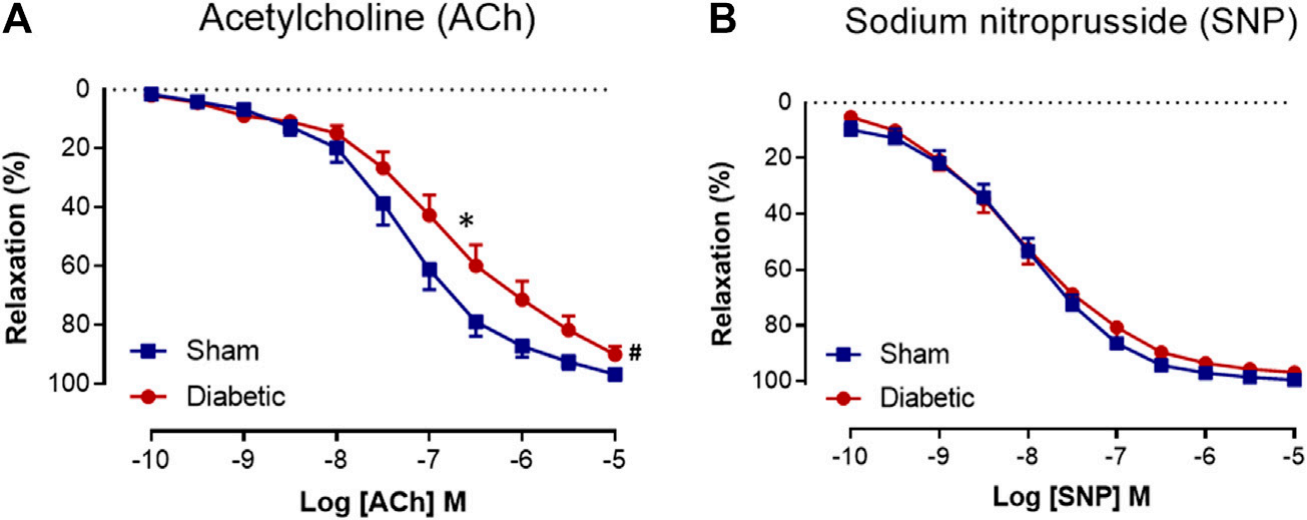
Cumulative concentration-response curves to ACh and SNP. Acetylcholine **(A)** (*n* = 14–16) and sodium nitroprusside **(B)** (*n* = 14–16) in isolated carotid arteries from sham (■) and diabetic (●) rats. Indomethacin (10 µM) was always present in the Krebs’ buffer. Values are shown as mean ± SEM, where *n* = number of animals. pEC_50_ *p < 0.05 vs sham animals, R_max_ #*p* < 0.05 vs sham animals (Unpaired Student’s *t*-test). See Table 1 for pEC_50_ and R_max_ values.

Relaxation responses to ACh and SNP in carotid arteries from sham and diabetic rats are shown in Figure 2. Diabetes significantly reduced the sensitivity and maximal relaxation to ACh compared to sham (Figure 2A, Table 1). However, the relaxation to the endothelium-independent vasodilator SNP was not significantly different between the two groups (Figure 2B, Table 1), suggesting that there was a selective impairment of endothelial function in the carotid arteries from diabetic rats.

**TABLE 1:** Pharmacological parameters of endothelial function *ex vivo*. A comparison of sensitivity (pEC_50_) and maximum relaxation (R_max_) to ACh or SNP in the absence or presence of various inhibitors in endothelium-intact carotid arteries isolated from sham and diabetic rats. All experiments were conducted in the presence of indomethacin (10 μmol/L). n = the number of experiments. Results are given as mean ± SEM. *p < 0.05 Vs. sham, one-way ANOVA with Dunnett’s post-hoc test. #*p* < 0.05,####*p* < 0.0001 vs. control within each group, one-way ANOVA with Dunnett’s test. ND: not determined.

ACh	n	Sham		n	Diabetic	
		pEC_50_	*R*_max_ (%)		pEC_50_	*R*_max_ (%)
Control	14	7.24 ± 0.15	96 ± 1	16	6.72 ± 0.18*	90 ± 3*
Apamin + TRAM 34	13	6.82 ± 0.22	93 ± 2	16	6.43 ± 0.16	88 ± 3
L-NAME + ODQ	6	ND	22 ± 7^####^	7	ND	17 ± 8^####^
Apamin + TRAM 34 + L-NAME + ODQ	6	ND	9 ± 6^####^	5	ND	13 ± 8^####^
Apamin + TRAM 34 + L-cysteine	6	6.77 ± 0.66	62 ± 9^####^	8	6.55 ± 0.26	60 ± 12^####^
Apamin + TRAM 34 + HXC	7	6.50 ± 0.17	72 ± 11^#^	8	6.44 ± 0.23	84 ± 6
SNP	**n**	**Sham**		**n**	**Diabetic**	
		pEC_50_	*R*_max_ (%)		pEC_50_	*R*_max_ (%)
Control	6	8.10 ± 0.18	98 ± 1	7	8.15 ± 0.11	97 ± 1
Apamin + TRAM 34	6	8.12 ± 0.28	100 ± 1	5	7.97 ± 0.11	95 ± 3
L-NAME + ODQ	6	ND	18 ± 8^####^	7	ND	28 ± 10^####^
Apamin + TRAM 34 + L-NAME + ODQ	6	ND	17 ± 8^####^	5	ND	41 ± 9^####^
Apamin + TRAM 34 + L-cysteine	6	6.65 ± 0.21^####^	87 ± 9	8	6.89 ± 0.19^####^	97 ± 2
Apamin + TRAM 34 + HXC	7	6.31 ± 0.21^####^	97 ± 1	8	6.86 ± 0.15^####^	97 ± 1

### Impact of Diabetes on the Relative Contribution of EDH and NOS-Derived Nitrogen Oxide Species to ACh-Evoked Relaxation

**FIGURE 3:**
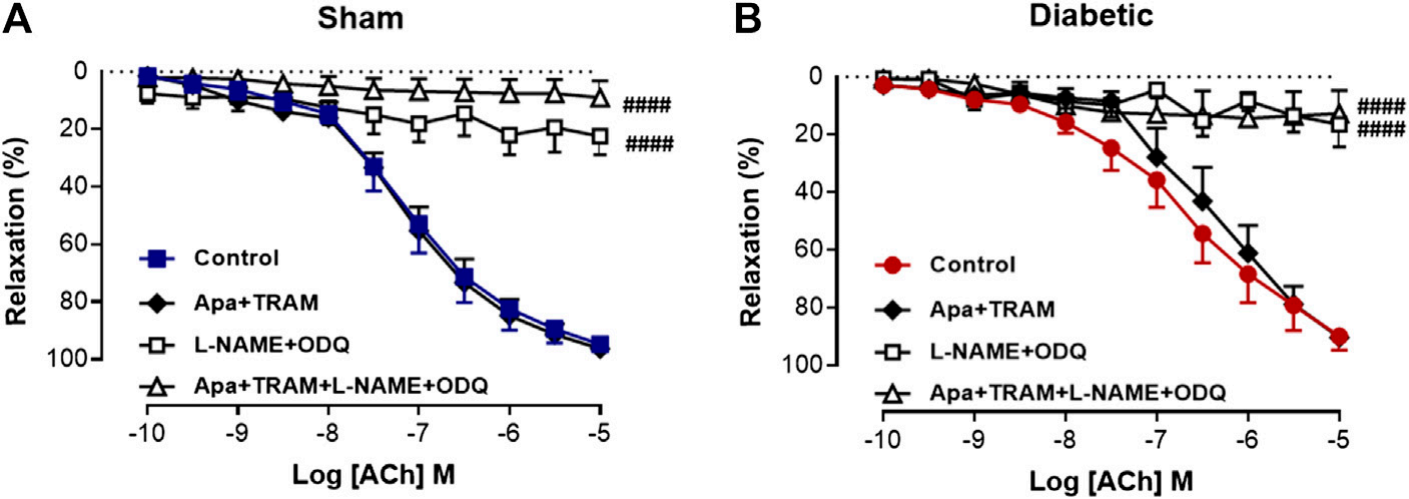
Relative contribution of EDH and NOS-derived nitrogen species to ACh-induced Relaxation in rat carotid arteries. Cumulative concentration response curves to ACh in the absence (control) and presence of either apamin + TRAM 34 (*n* = 6), L-NAME + ODQ (*n* = 6–7) and apamin + TRAM 34 + L-NAME + ODQ (*n* = 6). Carotid arteries isolated from **(A)** Sham **(B)** Diabetic rats. Indomethacin (10 µM) was always present in the Krebs’ buffer. Values are mean ± SEM, where *n* = number of animals. ####R_max_ vs control (*p* < 0.0001, one-way ANOVA, Dunnett’s *post-hoc* test). See Table 1 for pEC_50_ and R_max_ values.

Vascular reactivity to ACh was further assessed in the presence of either the combination of small and intermediate conductance Ca^2+^ activated K^+^ channel blockers (apamin and TRAM-34, respectively), or the combination of L-NAME and ODQ to determine the relative contribution of EDH and NOS-derived nitrogen oxide species, respectively, to endothelium-dependent relaxation in sham and diabetic rat carotid arteries (Figure 3, Table 1). In carotid arteries from both sham and diabetic groups, ACh-induced relaxation was not affected by the presence of apamin and TRAM-34, but virtually abolished by the combination of L-NAME and ODQ, indicating that a NOS-derived nitrogen oxide species is the predominant endothelium-derived vasodilator in carotid arteries rather than EDH (Figures 3A,B, Table 1). Furthermore, in carotid arteries from all groups, the relaxation response to ACh was completely abolished in the presence of apamin, TRAM-34, L-NAME and ODQ combined, which suggests that there was no contribution of non-nitrogen oxide species/non-EDH to relaxation (Figures 3A,B, Table 1).

### Effect of Diabetes on the Relative Contribution of Endothelium-Derived NO• and Nitroxyl to Endothelium-Dependent Relaxation

**FIGURE 4:**
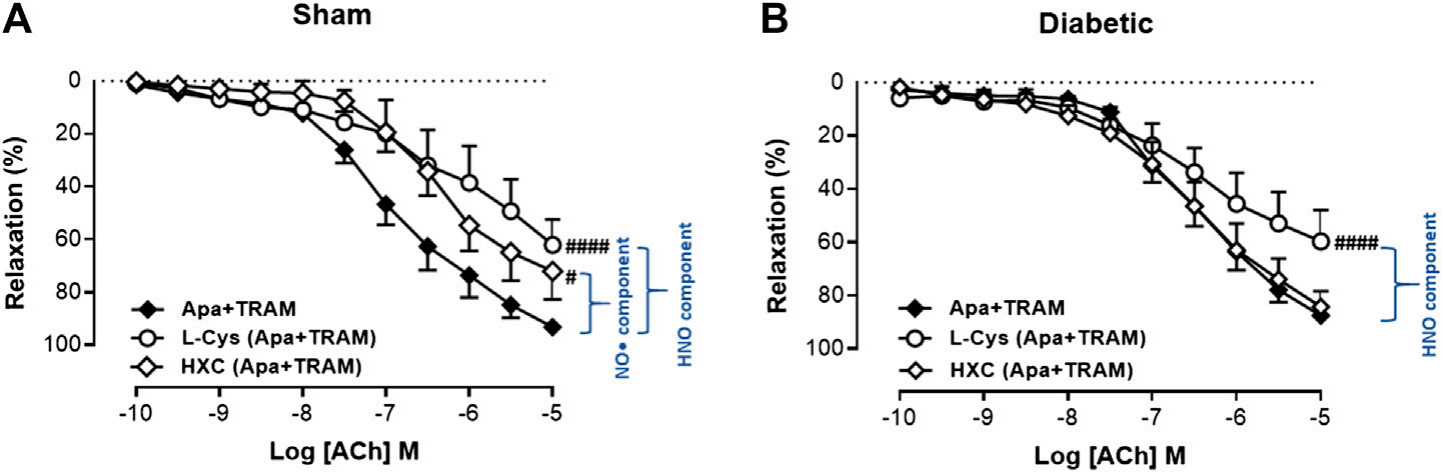
Relative contribution of NO• and HNO to ACh-induced relaxation in rat carotid arteries. Cumulative concentration response curves to ACh in the presence of apamin + TRAM 34 plus either L-cysteine (*n* = 7–8) or HXC (*n* = 8). Carotid arteries isolated from **(A)** Sham **(B)** Diabetic rats. Indomethacin (10 µM) was always present in the Krebs’ buffer. Values are mean ± SEM, where *n* = number of animals. R_max_ vs control (#*p* < 0.05, ####*p* < 0.0001, one-way ANOVA, Dunnett’s *post-hoc* test). See Table 1 for pEC_50_ and R_max_ values.

In order to determine the contribution of endothelium-derived NO• vs. HNO to relaxation in the carotid artery, the EDH component of endothelium-dependent relaxation was eliminated with K_Ca_ blockers (apamin + TRAM-34). Under these conditions, the relaxant response to ACh in the presence of HXC (NO• scavenger) or L-cysteine (HNO scavenger) is mediated by HNO or NO•, respectively. In sham carotid artery, the maximum relaxation response to ACh was significantly attenuated either by the presence of the NO• scavenger, HXC or the HNO scavenger, L-cysteine (Figure 4A, Table 1). Thus NO• and HNO both contributed to endothelium-dependent relaxation in the carotid arteries from sham rats. In contrast, in carotid arteries from diabetic rats, there was a significant decrease in the R_max_ to ACh in the presence of the HNO scavenger, L-cysteine, but not the NO• scavenger, HXC (Figure 4B, Table 1), indicating that NO•-mediated endothelium-dependent relaxation was impaired by diabetes whereas HNO-mediated relaxation was intact.

### Impact of Diabetes on Basal Levels of Nitrogen Oxide/NO•/Nitroxyl Bioavailability

**FIGURE 5:**
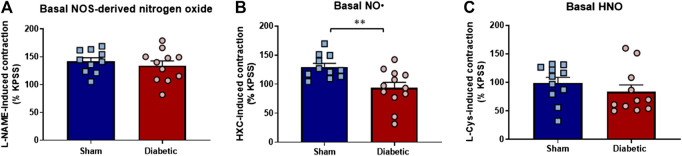
Basal level of nitrogen oxide, NO• and HNO bioavailability *ex vivo*. Diabetes did not affect maximum contraction to the eNOS inhibitor L-NAME **(A)**, which negates both NO• and HNO, but was associated with loss in endogenous bioavailability of **(B)** NO• (HXC-induced contraction) **(C)** Basal HNO bioavailability (L-NAME-induced contraction) was maintained in diabetic rat carotid arteries. Carotid arteries isolated from sham (blue bar) and diabetic (red bar) rats. Indomethacin (10 µM) was always present in the Krebs’ buffer. Results are shown as mean ± SEM, where *n* = 11–12 per group. **p < 0.005 vs sham rats (Unpaired Student’s *t*-test).

Diabetes did not affect the maximum contraction to KPSS (125 mmol/L) (Supplementary Figure S1). Similarly, L-NAME induced arterial contraction, attributed to the basal release of nitrogen oxides, was not significantly different between the two groups (Figure 5A). In contrast, the HXC-induced contraction, which reflected the basal level of NO•, was significantly reduced in carotid arteries from diabetic compared to sham rats (Figure 5B, *p* < 0.005). Furthermore, the contractile responses to L-cysteine, attributed to the basal level of HNO, was not significantly different in carotid arteries from diabetic compare to sham rats (Figure 5C). Together these findings suggest that the basal activity of endogenous NO• is reduced by diabetes whereas the basal release of HNO is preserved.

### Effect of Diabetes on the Relative Contribution of NO• and Nitroxyl to Sodium Nitroprusside-Induced Relaxation

**FIGURE 6:**
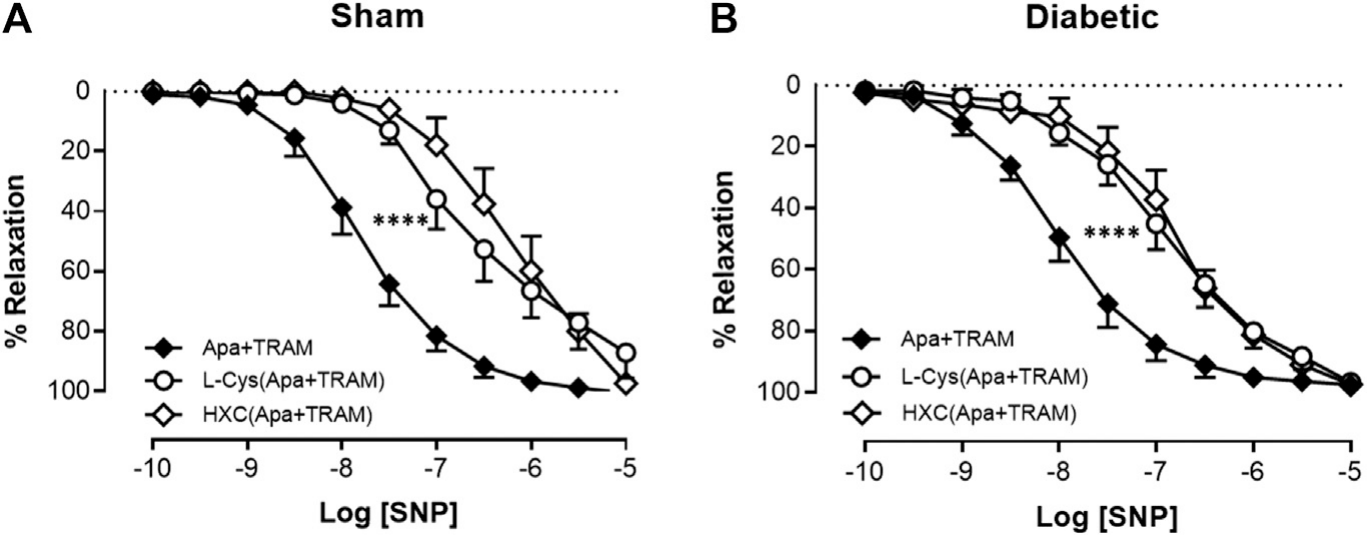
Relative contribution of NO• and HNO to SNP-induced relaxation in rat carotid arteries. Cumulative concentration response curves to SNP in the presence of apamin + TRAM 34 plus either L-cysteine (*n* = 6–8) or HXC (*n* = 7–8). Carotid arteries isolated from **(A)** Sham **(B)** Diabetic rats. Indomethacin (10 µM) was always present in the Krebs’ buffer. Values are mean ± SEM, where *n* = number of animals. pEC_50_ vs control (*p < 0.05, ****p < 0.0001, one-way ANOVA, Dunnett’s *post-hoc* test). See Table 1 for pEC_50_ and R_max_ values.

As indicated previously, diabetes had no the effect on relaxation response to SNP (Figure 2B, Table 1). The relative contribution of NO• and HNO to the endothelium-independent, SNP-induced relaxation in the carotid artery was examined. Similar to the assessment of endothelium-dependent relaxation, the EDH component was eliminated with KCa blockers (apamin + TRAM-34). The sensitivity, but not maximum relaxation response was significantly impaired by the presence of either a NO• scavenger (HXC with K_Ca_ blockers) or a HNO scavenger (L-cysteine with K_Ca_ blockers) in carotid arteries from both sham and diabetic rats (Figures 6A,B, Table 1), which demonstrated that both NO• and HNO contribute to relaxant responses to SNP and diabetes did not impair the responses to either of these mediators when released from SNP rather than the endothelium.

**FIGURE 7:**
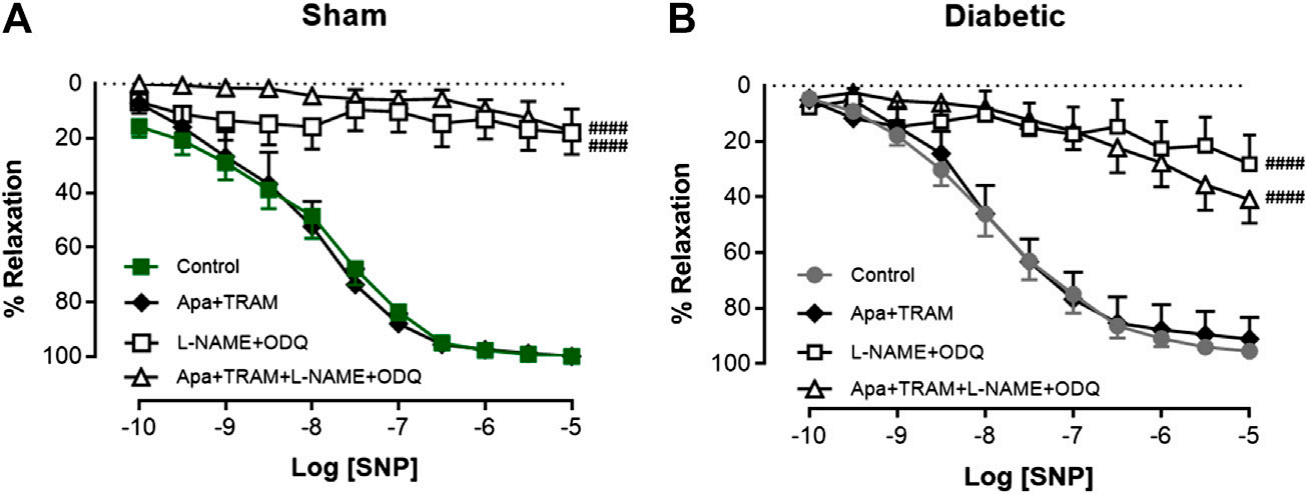
Relative contribution of SNP-induced relaxation in rat carotid arteries. Cumulative concentration response curves to SNP in the absence (control) and presence of either apamin + TRAM 34 (*n* = 5–6), L-NAME + ODQ (*n* = 6–7) or apamin + TRAM 34 + L-NAME + ODQ (*n* = 5–6). Carotid arteries isolated from **(A)** Sham **(B)** Diabetic rats. Indomethacin (10 µM) was always present in the Krebs’ buffer. Values are mean ± SEM, where *n* = number of animals. R_max_ vs control (^#^ *p* < 0.05, one-way ANOVA, Dunnett’s *post-hoc* test). See Table 1 for pEC_50_ and R_max_ values.

We then looked the contribution of sGC in SNP-mediated relaxation in carotid arties from sham or diabetic vessels. The response to SNP could almost be abolished either by the presence of L-NAME + ODQ or the presence of apamin, TRAM-34, L-NAME and ODQ combined (Figure 7A, Table 1) in carotid arteries from sham and diabetic rats (Figure 7B, Table 1), indicating that nitrogen oxide species largely acts on sGC.

## Discussion

**FIGURE 8:**
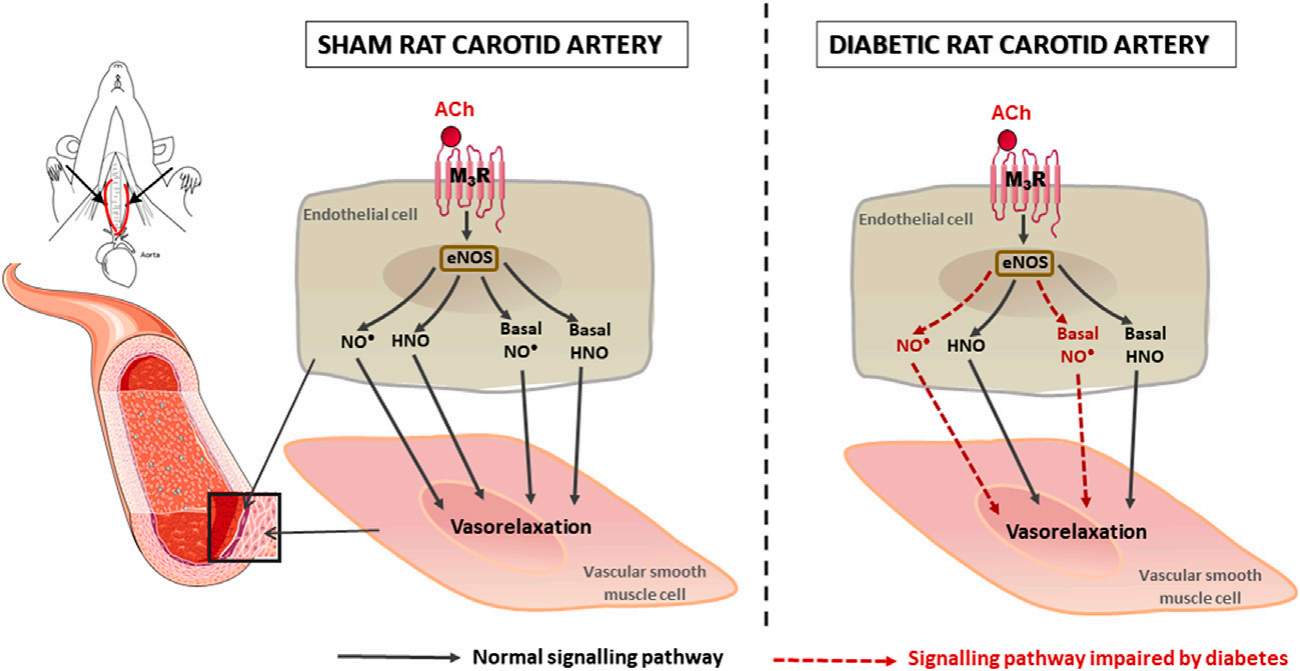
Schematic diagram to summarize the impact of diabetes on rat carotid artery function. Both NO• and HNO contribute to endothelium-dependent relaxation in carotid arteries. In diabetes, NO•-mediated relaxation is impaired, whereas HNO-mediated relaxation was preserved.

Diabetes is a known risk factor for stroke, a component of stroke risk is likely associated with diabetes-induced endothelial dysfunction (Sitia et al., 2010; Douglas and Channon, 2014). In this study, the endothelial function of carotid arteries, as well as the endothelium-derived nitrogen oxide-mediated relaxation was assessed in diabetic rats. Rats that received the HFD and two low-doses of STZ exhibited hyperglycaemia, elevated HbA_1c_ and reduced glucose tolerance. Endothelial dysfunction was evident in the carotid artery of diabetic rats, which was mainly due to a decreased contribution of NO•-mediated relaxation, whereas the contribution of HNO was maintained. There was no evidence of an EDH contribution to endothelium-dependent relaxation of carotid arteries from either control or diabetic rats. Diabetes also caused a decrease in basal NO• bioavailability in carotid arteries, but the basal HNO release was preserved (Figure 8). Our study demonstrated for the first time that although there was selective impairment of endothelial function, the stimulated and basal release of HNO was preserved in the carotid artery of diabetic animals.

T2DM is a complex metabolic disorder essentially characterized by insulin resistance and a defect in pancreatic β-cell mass and function, and that is strongly influenced by lifestyle and diet (Guilherme et al., 2008; Podell et al., 2017; Ritchie and Abel, 2020). Previous studies have indicated that an animal model that incorporates an HFD to induce peripheral insulin resistance (Sankar et al., 2012; Podell et al., 2017; Sampath et al., 2017), is often associated with limited end-organ damage. Additional low dose STZ administration with HFD only destroys a portion of (not all) the pancreatic β-cells to increase plasma glucose moderately. This phenotype closely mimics the pathogenesis of human T2DM (Reed et al., 2000; Asrafuzzaman et al., 2017). The HFD + low dose STZ approach has been gaining popularity in recent years, providing an alternative to the existing genetic model of T2DM (e.g. *db/db* mice, Zucker rats). This model was first reported by the Reed group (Reed et al., 2000), where they have demonstrated that rats that were fed an HFD exhibited high blood insulin levels but essentially normal blood glucose concentrations. The additional low dose of STZ injection led to mild impairment in insulin secretion, closely resembling the key characteristics of insulin resistance and pancreatic β-cell dysfunction in human T2D. Since then, different combinations of diet and STZ dosage have been developed.

In the present study, we have adopted a model similar to Marsh et al. (Marsh et al., 2009), using a combination of HFD (SF03-002; total digestible energy: 59% lipids, 15% protein; wt/wt: 34.6% sucrose; Specialty Feeds, WA, Australia) and two low-dose injections of STZ (30 mg/kg, 24-h apart). Our model of HFD/STZ rats exhibited hyperglycaemia and elevated HbA1c, which was apparent as early as 2 weeks following the second injection of STZ. Accompanying this hyperglycaemia, the rats displayed many characteristics of T2DM including reduced glucose tolerance and retarded weight gain, which are consistent with previous studies where an HFD was combined with multiple low-doses of STZ in rats (Zhang et al., 2008; Albersen et al., 2011). The combination of elevated plasma glucose but with a maintained plasma insulin level in these HFD/STZ rats is consistent with insulin resistance (Reed et al., 2000; Sharma et al., 2011; Guo et al., 2012). However, the lower body weight and fat mass at study endpoint compared to controls is not entirely reflective of obese T2DM (Lin et al., 2018; Bai et al., 2019), but rather as a model of T2DM, an emerging clinical feature in many diabetic populations (Balasubramanyam et al., 2011; Florez and Castillo-Florez, 2012).

T2DM-associated vascular dysfunction is a major clinical problem that is linked with a higher incidence of coronary artery, peripheral vascular and microvascular disease (Laakso, 1999; Eleftheriadou et al., 2019). Endothelial dysfunction has been defined as a common biomarker of diabetes (Iellamo et al., 2006; Vanhoutte et al., 2017), with compromised signaling of endothelium-derived relaxing factors playing a major role. Endothelial dysfunction has been well-characterized in the STZ-induced model of experimental type 1 diabetes mellitus (T1DM) (Makino et al., 2000; Matsumoto et al., 2003) as well as in *db/db* mice, which are spontaneously diabetic as a result of inherited gene mutation (Pannirselvam et al., 2002; Gao et al., 2010; Lee et al., 2011; Park et al., 2011). In the present study, carotid arteries isolated from HFD plus STZ-induced diabetic rats exhibited impaired endothelium-dependent relaxation in response to ACh. By contrast, the response to the endothelium-independent vasodilator, SNP, was not affected by diabetes, indicating that vascular smooth muscle function was intact. Thus, endothelial function was selectively impaired by diabetes, suggesting that endothelial cells are more vulnerable than smooth muscle cells to diabetes-induced impairment. These observations are consistent with other studies that have demonstrated that diabetes causes endothelial dysfunction but does not affect smooth muscle function, in either conduit or resistance vasculature (Leo et al., 2011; Kahlberg et al., 2016).

In order to evaluate nitrogen oxide-mediated relaxation, EDH-induced relaxation was inhibited by IK_Ca_ and SK_Ca_ channel blockers, apamin and TRAM-34, respectively. Thus, the residual vascular relaxation response to ACh was attributed to nitrogen oxide species (i.e. NO• and HNO). We observed here that endothelium-dependent relaxation was not affected by the presence of K_Ca_ blockers in either sham or diabetic carotid arteries, indicating that endothelium-derived nitrogen oxide species are the main contributors to endothelium-dependent vasodilatation in large conduit carotid arteries. This observation is consistent with several other studies (Joannides et al., 1995; Woodman et al., 2000; Takaki et al., 2008; Feletou et al., 2012).

In addition, similar observations have been made in the SNP-mediated relaxation in the current study. We found that the vasorelaxation response to SNP was only affected by the presence of the NOS inhibitor plus sGC inhibitor (L-NAME + ODQ) in both sham and diabetic carotid arteries, demonstrating that nitrogen oxide species acting on sGC are the predominant dilator mechanisms of SNP-induced relaxation in the carotid arteries.

To examine whether EDH contributed to endothelium-dependent relaxation of carotid arteries in diabetes, the combination of L-NAME and ODQ were used to inhibit NOS and sGC activities, respectively. The presence of L-NAME and ODQ (and the cyclooxygenase inhibitor-indomethacin) abolished the ACh-induced vasodilatation response in diabetic as well as sham rats, but not the K_Ca_ blockers, indicating that EDH does not contribute to endothelium-dependent relaxation in carotid arteries. It has been reported that the contribution of EDH to endothelium-dependent relaxation is predominantly found in resistance arteries (i.e. small arteries with diameters of less than 500 μM) (Mulvany and Aalkjær, 1990; Tomioka et al., 1999; Shimokawa and Godo, 2016; Garland and Dora, 2017) and more likely to be preserved, or even upregulated, to compensate for the loss of NO•-mediated relaxation in a disease state, such as diabetes (Cho et al., 2013; Kobuchi et al., 2015; Mokhtar et al., 2016a; Mokhtar et al., 2016b). Whereas it has been reported that EDH may play a role in the carotid in diabetes as there is increased expression of IKCa and a contribution of EDH early in the disease process in carotid arteries from diabetic animals (Leo et al., 2010; Centeno et al., 2019). In contrast, in this study with more advanced diabetes there was no compensation from EDH for diabetes-induced impairment of endothelium-dependent relaxation in the carotid artery, which is consistent with a previous study (Shi et al., 2006).

To investigate the potential role of endogenous HNO as an endothelium-derived vasodilator in carotid arteries, we employed the well-characterized pharmacological tools, L-cysteine (HNO scavenger) (Pino and Feelisch, 1994; Ellis et al., 2000; Irvine et al., 2003; Andrews et al., 2009) and HXC (NO• scavenger) (Li and Rand, 1993; Wanstall et al., 2005; Andrews et al., 2009). In the current study, we provide evidence that both endogenous, eNOS-derived NO• and HNO exist in carotid arteries from sham or diabetic rats. These findings confirm that the endogenous eNOS-derived NO• and HNO are both released in the conduit vasculature. This observation is consistent with a recent study that demonstrated that there was endogenous eNOS-derived nitrogen oxide (NO• and HNO) present in resistance vessels (Kahlberg et al., 2016).

We demonstrated that in the presence of either HXC or L-cysteine, the maximal relaxation response to ACh was decreased when compared to control arteries. Such observations indicate that both NO• and HNO contribute to ACh-induced relaxation in carotid arteries from sham rats. The HNO scavenger L-cysteine also attenuated ACh-induced maximal relaxation in the diabetic vasculature, whereas the NO• scavenger HXC did not. Further, we observed a significant reduction in the basal level of NO• as a result of diabetes evidenced by a significant reduction in HXC-induced contraction in carotid arteries from diabetic compared to sham rats. In contrast, there was no difference in the basal level of HNO between the sham and diabetic group as L-cysteine-induced contraction was not changed. Together these findings suggest that the bioavailability of endogenous NO• and NO•-mediated relaxation was impaired in diabetes, whereas the component of HNO is preserved in the context of experimental diabetes. Additionally, as mentioned previously, there was a significant reduction in the basal release of NO• as a result of diabetes. In contrast, there was no difference in the contractile response to either inhibition of basal HNO or nitrogen oxide release between the two groups. This may be due HNO scavenger that was employed in the present study (i.e. L-cysteine), which may not only attenuate HNO but also enhance and prolong the actions of NO• (Pino and Feelisch, 1994; Ellis et al., 2000; Wanstall et al., 2001) and thus may increase endogenous NO• bioavailability along with a loss of endogenous HNO.

The major mechanism implicated in diabetes-induced endothelial dysfunction includes increased oxidative stress and compromised NO• signaling (De Vriese et al., 2000; Fatehi-Hassanabad et al., 2010; Leo et al., 2010; Leo et al., 2011). The impairment of NO•-mediated relaxation in the diabetic vasculature occurs through several pathways including a deficiency in L-arginine or BH_4_ levels. This critical substrate and cofactor, respectively, are required for NO• synthesis by eNOS (Boucher et al., 1999; Luiking et al., 2010). Decreased levels of L-arginine or BH_4_ can lead to uncoupling of eNOS, which consequently results in the generation of ROS instead of NO• in the vasculature (Chang et al., 1993; Beckman and Koppenol, 1996). ROS such as •O_2_^−^ reacts rapidly with NO• to produce peroxynitrite (ONOO^─^) (Paolocci et al., 2001), simultaneously increasing ONOO^─^ -induced cellular toxicity (Beckman and Koppenol, 1996; Beckman and Zu Ye, 1996). It has been reported that peroxynitrite can lead to eNOS uncoupling through oxidation of BH_4_ to dihydrobiopterin (BH_2_), thus further promoting overproduction of •O_2_^−^ (Zou et al., 2004). This overproduction of •O_2_^−^ is likely an early feature of diabetic vascular disease, which contributes to impaired NO• synthesis/activity and endothelial dysfunction (Guzik et al., 2002; Pennathur and Heinecke, 2007; Velagic et al., 2020). There is an accumulating body of evidence to demonstrate that NO•-mediated relaxation is impaired in diabetes. For example, several studies have demonstrated that endothelial dysfunction is associated with a significant reduction in endothelium-dependent NO•-mediated relaxation in diabetic mesenteric arteries which was caused by increased oxidative stress (Makino et al., 2000; Matsumoto et al., 2003; Leo et al., 2011). In addition, in the *db/db* mouse model of T2DM, impaired NO•-mediated vasodilatation was observed in the coronary vasculature (Park et al., 2008).

Previous studies have also demonstrated that the contribution of HNO to endothelium-dependent relaxation is preserved in arteries from hypertensive mice (Wynne et al., 2012), T1DM rats (Leo et al., 2012; Kahlberg et al., 2016; Tare et al., 2017) and hypercholesterolaemic mice (Bullen et al., 2011; Jelinic et al., 2014) where oxidative stress is also evident. Pharmacological studies have provided evidence to suggest that HNO is resistant to scavenging by •O_2_^−^ and lacks reactivity with •O_2_^−^ to form ONOO^−^ (Miranda et al., 2002; Leo et al., 2012), thus resulting in preserved HNO-mediated vasodilatation (Miranda et al., 2002). Furthermore, HNO itself can limit •O_2_^−^ production by directly inhibiting vascular NADPH oxidase (Nox2) (Miller et al., 2009), supporting our results that endogenous generation and/or bioavailability of HNO is preserved in the large conduit artery in this model of diabetes. Our demonstration that, in the rat carotid artery, diabetes impairs responses to endogenous NO• whereas endogenous HNO activity is preserved provides further support to the proposal that HNO donors may prove more effective than classical NO• donors in the treatment of cardiovascular disease (Andrews et al., 2016; Chin et al., 2016; Qin et al., 2020). It has been previously demonstrated that in diabetes, and other causes of oxidative stress, there is impairment of vascular and cardiac responses to NO• donors (Van Etten et al., 2002; Okon et al., 2005; Shemyakin et al., 2012; Qin et al., 2020) referred to as NO• resistance. Further, while tolerance to the chronic use of NO• donors is a well-established phenomenon, HNO donors maintain their efficacy with continued use (Irvine et al., 2007; Kemp-Harper, 2011; Dautov et al., 2013; Tare et al., 2017). Efforts are underway to develop new generation HNO donors with optimal half-lives to enhance their therapeutic utility (Cowart et al., 2019). Although the current studies are directed toward their potential use in congestive heart failure. Our study suggests that it may be worthwhile investigating their vascular-protective effects in diabetes.

The relative contribution of NO• and HNO to SNP-induced relaxation was also investigated. Interestingly, we found that sensitivity to SNP in rat carotid arteries was impaired by both HXC and L-cysteine, indicating that both NO• and HNO can be released from SNP. This finding is in agreement with those of other investigators (Ellis et al., 2000; Irvine et al., 2003). In addition, there is no significant differences between sham and diabetic vessels for the same inhibitors used, suggesting that exogenous nitrogen oxide (either NO• or HNO) was not affected by diabetes, though endogenous nitrogen oxide could be reduced in the disease state. This observation strengthens the concept that the release of NO• from the endothelium is impaired by diabetes rather than the action of NO• on the smooth muscle.

### Limitations of the Study

Despite the findings obtained regarding the likely effect of diabetes on endogenous NO• and HNO-mediated relaxation and their basal level in carotid arteries, there are limitations of the current study that should be noted. In this study, we investigated the effect of L-cysteine and HXC individually on the relaxation response to ACh, but not the impact of both inhibitors in combination. While in our earlier studies, we have assessed the combined effects of HXC + L-cysteine in the rat aorta (Leo et al., 2012), femoral arteries (Tare et al., 2017) and mesenteric arteries (Kahlberg et al., 2016). In all vessels, the combination of HXC + L-Cysteine abolished NO/HNO mediated relaxation to a similar extent as NOS inhibition. Moreover, it is also important to note, however, that definitive evidence for endogenous production of HNO could not be acquired in the present study, since there remains a lack of validated methods to measure HNO at the tissue level (Kahlberg et al., 2016).

## Conclusion

In conclusion, we have confirmed that nitrogen oxide species (NO• and HNO) are the major contributors to endothelium-dependent relaxation in the rat carotid artery, with no contribution by EDH. Importantly, this study has demonstrated that diabetes impairs NO•-mediated vasorelaxation and basal release of NO•, while the HNO-mediated vasorelaxation is preserved, as is basal HNO release. The preservation of HNO bioavailability found in this study indicates that there is a potential for HNO donors to be employed as therapeutic agents for the treatment of vascular dysfunction associated with diabetes.

## Data Availability

The raw data supporting the conclusions of this article will be made available by the authors, without undue reservation, to any qualified researcher.

## Glossary

ACh: acetylcholine
BH_4_: tetrahydrobiopterin
Ca^2+^: calcium
COX: cyclooxygenase
DMSO: dimethyl sulfoxide
EDH: endothelium-dependent hyperpolarization
eNOS: endothelial nitric oxide synthase
GTT: glucose tolerance test
HFD: high fat diet
HNO: nitroxyl
HXC: hydroxocobalamin
IK_Ca_,: intermediate-conductance Ca^2+^-activated K^+^ channel
KCa,: calcium-activated potassium channel
KPSS,: high K^+^-containing physiological saline solution
L-Cys: L-cysteine
L-NAME: Nω -nitro- L-arginine methyl ester
NO•: nitric oxide
Nox2: NADPH oxidase 2
•O_2_‐: superoxide
ODQ: 1H-[1,2,4]oxadiazolo[4,3-a]quinoxalin-1-one
ONOO^–^: peroxynitrite
PE: phenylephrine
pEC_50_: negative log of half-maximal effective concentration
R_max_: maximum relaxation
ROS: reactive oxygen species
SD: Sprague Dawley
sGC: soluble guanylate cyclase
SK_Ca,_: small-conductance Ca^2+^-activated K^+^ channel
SNP: sodium nitroprusside
STZ: streptozotocin
T2DM: type 2 diabetes mellitus
TRAM-34: 1-[(2-chlorophenyl)(diphenyl)methyl]-1H-pyrazole
U46619 9,: 11-dideoxy-9α, 11α-methanoepoxy-PGF2α.

## References

[B1] Adak S. Wang Q. Stuehr D. J. (2000). Arginine conversion to nitroxide by tetrahydrobiopterin-free neuronal nitric-oxide synthase. Implications for mechanism. J. Biol. Chem. 275, 33554–33561. 10.1074/jbc.M004337200 10945985

[B2] Albersen M. Lin G. Fandel T. M. Zhang H. Qiu X. Lin C. S. (2011). Functional, metabolic, and morphologic characteristics of a novel rat model of type 2 diabetes-associated erectile dysfunction. Urology 78, 476–478. 10.1016/j.urology.2011.03.024 PMC3152638 21624647

[B3] Andrews K. L. Irvine J. C. Tare M. Apostolopoulos J. Favaloro J. L. Triggle C. R. (2009). A role for nitroxyl (HNO) as an endothelium-derived relaxing and hyperpolarizing factor in resistance arteries. Br. J. Pharmacol. 157, 540–550. 10.1111/j.1476-5381.2009.00150.x 19338582 PMC2707966

[B4] Andrews K. L. Sampson A. K. Irvine J. C. Shihata W. A. Michell D. L. Lumsden N. G. 2016). Nitroxyl (HNO) reduces endothelial and monocyte activation and promotes M2 macrophage polarization. Clin. Sci. 130, 1629–1640. 10.1042/CS20160097 27231254

[B5] Asrafuzzaman M. Cao Y. Afroz R. Kamato D. Gray S. Little P. J. (2017). Animal models for assessing the impact of natural products on the aetiology and metabolic pathophysiology of Type 2 diabetes. Biomed. Pharmacother. 89, 1242–1251. 10.1016/j.biopha.2017.03.010 28320091

[B6] Bai B. Yang W. Fu Y. Foon H. L. Tay W. T. Yang K. 2019). Seisin knockout mice develop heart failure with preserved ejection fraction. JACC Basic Transl Sci. 4, 924–937. 10.1016/j.jacbts.2019.07.008 31909301 PMC6939002

[B7] Balasubramanyam A. Yajnik C. S. Tandon N. (2011). Non-traditional forms of diabetes worldwide: implications for translational investigation. Translational Endocrinology and Metabolism. 2, 43–67. 10.1210/TEAM.9781879225824.ch2

[B8] Beckman J. S. Koppenol W. H. (1996). Nitric oxide, superoxide, and peroxynitrite: the good, the bad, and ugly. Am. J. Physiol. 271, C1424–C1437. 10.1152/ajpcell.1996.271.5.C1424 8944624

[B9] Beckman J. S. Ye Y. Chen J. Conger K. A. 1996). The interactions of nitric oxide with oxygen radicals and scavengers in cerebral ischemic injury. Adv. Neurol. 71, 339–344. 8790810

[B10] Boucher J. Moali C. Tenu J. (1999). Nitric oxide biosynthesis, nitric oxide synthase inhibitors and arginase competition for L-arginine utilization. Cell. Mol. Life Sci. 55, 1015–1028. 10.1007/s000180050352 10484661 PMC11147020

[B11] Bullen M. L. Miller A. A. Andrews K. L. Irvine J. C. Ritchie R. H. Sobey C. G. (2011). Nitroxyl (HNO) as a vasoprotective signaling molecule. Antioxidants Redox Signal. 14, 1675–1686. 10.1089/ars.2010.3327 20673125

[B12] Cade W. T. (2008). Diabetes-related microvascular and macrovascular diseases in the physical therapy setting. Phys. Ther. 88, 1322–1335. 10.2522/ptj.20080008 18801863 PMC2579903

[B13] Centeno J. M. López-Morales M. A. Aliena-Valero A. Jover-Mengual T. Burguete M. C. Castelló-Ruiz M. (2019). Potassium channels contribute to the increased sensitivity of the rabbit carotid artery to hydrogen sulfide in diabetes. Eur. J. Pharmacol. 853, 33–40. 10.1016/j.ejphar.2019.03.019 30876977

[B14] Chang K. Chung S. Chong W. Suh J. Kim S. Noh H. (1993). Possible superoxide radical-induced alteration of vascular reactivity in aortas from streptozotocin-treated rats. J. Pharmacol. Exp. Therapeut. 266, 992–1000. 8394927

[B15] Chin K. Y. Michel L. Qin C. X. Cao N. Woodman O. L. Ritchie R. H. (2016). The HNO donor Angeli's salt offers potential haemodynamic advantages over NO or dobutamine in ischaemia-reperfusion injury in the rat heart *ex vivo* . Pharmacol. Res. 104, 165–175. 10.1016/j.phrs.2015.12.006 26747404

[B16] Cho Y. E. Basu A. Dai A. Heldak M. Makino A. (2013). Coronary endothelial dysfunction and mitochondrial reactive oxygen species in type 2 diabetic mice. Am. J. Physiol. Cell Physiol. 305, C1033–C1040. 10.1152/ajpcell.00234.2013 23986204 PMC3840199

[B17] Coleman H. Tare M. Parkington H. (2017). Nonlinear effects of potassium channel blockers on endothelium-dependent hyperpolarization. Acta Physiol. 219, 324–334. 10.1111/apha.12805 27639255

[B18] Cowart D. Venuti R. P. Lynch K. Guptill J. T. Noveck R. J. Foo S. Y. (2019). A phase 1 randomized study of single intravenous infusions of the novel nitroxyl donor BMS‐986231 in healthy volunteers. J. Clin. Pharmacol. 59, 717–730. 10.1002/jcph.1364 30703258 PMC6519195

[B19] Dautov R. Ngo D. Licari G. Liu S. Sverdlov A. L. Ritchie R. H. (2013). The nitric oxide redox sibling nitroxyl partially circumvents impairment of platelet nitric oxide responsiveness. Nitric Oxide 35, 72–78. 10.1016/j.niox.2013.08.006 24012721

[B20] De Vriese A. S. Verbeuren T. J. Van De Voorde J. Lameire N. H. Vanhoutte P. M. (2000). Endothelial dysfunction in diabetes. Br. J. Pharmacol. 130, 963–974. 10.1038/sj.bjp.0703393 10882379 PMC1572156

[B21] Dempsey R. J. Vemuganti R. Varghese T. Hermann B. P. (2010). A review of carotid atherosclerosis and vascular cognitive decline: a new understanding of the keys to symptomology. Neurosurgery. 67, 484. 10.1227/01.NEU.0000371730.11404.36 20644437 PMC2908960

[B22] Ding S. Zhang M. Zhao Y. Chen W. Yao G. Zhang C. (2008). The role of carotid plaque vulnerability and inflammation in the pathogenesis of acute ischemic stroke. Am. J. Med. Sci. 336, 27–31. 10.1097/MAJ.0b013e31815b60a1 18626232

[B23] Douglas G. Channon K. M. (2014). The pathogenesis of atherosclerosis. Medicine. 42, 480–484.

[B24] Dutton A. S. Fukuto J. M. Houk K. (2004). Mechanisms of HNO and NO production from Angeli's salt: density functional and CBS-QB3 theory predictions. J. Am. Chem. Soc. 126, 3795–3800. 10.1021/ja0391614 15038733

[B25] Eleftheriadou I. Tentolouris A. Grigoropoulou P. Tsilingiris D. Anastasiou I. Kokkinos A. (2019). The association of diabetic microvascular and macrovascular disease with cutaneous circulation in patients with type 2 diabetes mellitus. J. Diabet. Complicat. 33, 165–170. 10.1016/j.jdiacomp.2018.10.008 30446479

[B26] Ellis A. Li C. Rand M. J. (2000). Differential actions of L-cysteine on responses to nitric oxide, nitroxyl anions and EDRF in the rat aorta. Br. J. Pharmacol. 129, 315–322. 10.1038/sj.bjp.0703058 10694238 PMC1571842

[B27] Endemann D. H. Schiffrin E. L. (2004). Endothelial dysfunction. J. Am. Soc. Nephrol. 15, 1983–1992. 10.1097/01.ASN.0000132474.50966.DA 15284284

[B28] Fatehi-Hassanabad Z. Chan C. B. Furman B. L. (2010). Reactive oxygen species and endothelial function in diabetes. Eur. J. Pharmacol. 636, 8–17. 10.1016/j.ejphar.2010.03.048 20371238

[B29] Félétou M. Köhler R. Vanhoutte P. M. (2012). Nitric oxide: orchestrator of endothelium-dependent responses. Ann. Med. 44, 694–716. 10.3109/07853890.2011.585658 21895549

[B30] Florez H. Castillo-Florez S. (2012). Beyond the obesity paradox in diabetes: fitness, fatness, and mortality. Jama. 308, 619–620. 10.1001/jama.2012.9776 22871873

[B31] Fowler M. J. (2008). Microvascular and macrovascular complications of diabetes. Clin. Diabetes 26, 77–82. 10.4103/2230-8210.183480

[B32] French W. Dridi S. Shouse S. Wu H. Hawley A. Lee S. O. (2017). A high-protein diet reduces weight gain, decreases food intake, decreases liver fat deposition, and improves markers of muscle metabolism in obese Zucker rats. Nutrients 9, 587. 10.3390/nu9060587 PMC5490566 28594375

[B33] Fukuto J. M. Wallace G. C. Hszieh R. Chaudhuri G. (1992). Chemical oxidation of N-hydroxyguanidine compounds. Release of nitric oxide, nitroxyl and possible relationship to the mechanism of biological nitric oxide generation. Biochem. Pharmacol. 43, 607–613. 10.1016/0006-2952(92)90584-6 1540216

[B34] Gao X. Picchi A. Zhang C. (2010). Upregulation of TNF-alpha and receptors contribute to endothelial dysfunction in zucker diabetic rats. Am J Biomed Sci. 2, 1. 10.5099/aj100100001 20559450 PMC2886289

[B35] Garland C. Dora K. (2017). EDH: endothelium-dependent hyperpolarization and microvascular signalling. Acta Physiol. 219, 152–161. 10.1111/apha.12649 26752699

[B36] Genc S. Omer B. Aycan-Ustyol E. Ince N. Bal F. Gurdol F. (2012). Evaluation of turbidimetric inhibition immunoassay (TINIA) and HPLC methods for glycated haemoglobin determination. J. Clin. Lab. Anal. 26, 481–485. 10.1002/jcla.21550 23143632 PMC6807436

[B37] Guilherme A. Virbasius J. V. Puri V. Czech M. P. (2008). Adipocyte dysfunctions linking obesity to insulin resistance and type 2 diabetes. Nat. Rev. Mol. Cell Biol. 9, 367–377. 10.1038/nrm2391 18401346 PMC2886982

[B38] Guo Z. Qin Z. Zhang R. Li J. Yin Y. (2012). Effect of rosiglitazone on the expression of cardiac adiponectin receptors and NADPH oxidase in type 2 diabetic rats. Eur. J. Pharmacol. 685, 116–125. 10.1016/j.ejphar.2012.04.010 22542658

[B39] Guzik T. J. Mussa S. Gastaldi D. Sadowski J. Ratnatunga C. Pillai R. (2002). Mechanisms of increased vascular superoxide production in human diabetes mellitus: role of NAD(P)H oxidase and endothelial nitric oxide synthase. Circulation 105, 1656–1662. 10.1161/01.cir.0000012748.58444.08 11940543

[B40] Hickman D. L. Johnson S. W. (2011). Evaluation of the aesthetics of physical methods of euthanasia of anesthetized rats. J Am Assoc Lab Anim Sci. 50, 695–701. 22330717 PMC3189674

[B41] Iellamo F. Tesauro M. Rizza S. Aquilani S. Cardillo C. Iantorno M. (2006). Concomitant impairment in endothelial function and neural cardiovascular regulation in offspring of type 2 diabetic subjects. Hypertension 48, 418–423. 10.1161/01.HYP.0000234648.62994.ab 16864746

[B42] Irvine J. C. Favaloro J. L. Kemp-Harper B. K. (2003). NO- activates soluble guanylate cyclase and Kv channels to vasodilator resistance arteries. Hypertension 41, 1301–1307. 10.1161/01.HYP.0000072010.54901.DE 12743008

[B43] Irvine J. C. Favaloro J. L. Widdop R. E. Kemp-Harper B. K. (2007). Nitroxyl anion donor, Angeli's salt, does not develop tolerance in rat isolated aortae. Hypertension 49, 885–892. 10.1161/01.HYP.0000259328.04159.90 17309955

[B44] Jelinic M. Leo C. H. Post Uiterweer E. D. Sandow S. L. Gooi J. H. Wlodek M. E. (2014). Localization of relaxin receptors in arteries and veins, and region-specific increases in compliance and bradykinins-mediated relaxation after *in vivo* serelaxin treatment. Faseb. J. 28, 275–287. 10.1096/fj.13-233429 24036884 PMC3868828

[B45] Joannides R. Haefeli W. E. Linder L. Richard V. Bakkali E. H. Thuillez C. (1995). Nitric oxide is responsible for flow-dependent dilatation of human peripheral conduit arteries *in vivo* . Circulation 91, 1314–1319. 10.1161/01.cir.91.5.1314 7867167

[B46] Kagota S. Chia E. Mcguire J. J. (2011). Preserved arterial vasodilatation via endothelial protease-activated receptor-2 in obese type 2 diabetic mice. Br. J. Pharmacol. 164, 358–371. 10.1111/j.1476-5381.2011.01356.x 21426317 PMC3174416

[B47] Kahlberg N. Qin C. X. Anthonisz J. Jap E. Ng H. H. Jelinic M. (2016). Adverse vascular remodelling is more sensitive than endothelial dysfunction to hyperglycaemia in diabetic rat mesenteric arteries. Pharmacol. Res. 111, 325–335. 10.1016/j.phrs.2016.06.025 27363948

[B48] Kamata K. Ohuchi K. Kirisawa H. (2000). Altered endothelium-dependent and -independent hyperpolarization and endothelium-dependent relaxation in carotid arteries isolated from streptozotocin-induced diabetic rats. Naunyn-Schmiedeberg’s Arch. Pharmacol. 362, 52–59. 10.1007/s002100000248 10935533

[B49] Kemp-Harper B. K. (2011). Nitroxyl (HNO): a novel redox signaling molecule. NY 10801 USA: Mary Ann Liebert, Inc. 10.1089/ars.2011.3937 21299468

[B50] Kobuchi S. Miura K. Iwao H. Ayajiki K. (2015). Nitric oxide modulation of endothelium-derived hyperpolarizing factor in agonist-induced depressor responses in anesthetized rats. Eur. J. Pharmacol. 762, 26–34. 10.1016/j.ejphar.2015.04.053 25962662

[B51] Kwee R. M. Van Oostenbrugge R. J. Mess W. H. Prins M. H. Van Der Geest R. J. Ter Berg J. W. 2013). MRI of carotid atherosclerosis to identify TIA and stroke patients who are at risk of a recurrence. J. Magn. Reson. Imag. 37, 1189–1194. 10.1002/jmri.23918 23166040

[B52] Laakso M. (1999). Hyperglycemia and cardiovascular disease in type 2 diabetes. Diabetes 48, 937–942. 10.2337/diabetes.48.5.937 10331395

[B53] Lee S. Park Y. Zhang C. (2011). Exercise training prevents coronary endothelial dysfunction in type 2 diabetic mice. Am J Biomed Sci. 3, 241. 10.5099/aj110400241 22384308 PMC3289260

[B54] Leo C. H. Joshi A. Woodman O. L. (2010). Short-term type 1 diabetes alters the mechanism of endothelium-dependent relaxation in the rat carotid artery. Am. J. Physiol. Heart Circ. Physiol. 299, H502–H511. 10.1152/ajpheart.01197.2009 20543087

[B55] Leo C. H. Ng H. H. Marshall S. A. Jelinic M. Rupasinghe T. Qin C. (2020). Relaxin reduces endothelium-derived vasoconstriction in hypertension: revealing new therapeutic insights. Br. J. Pharmacol. 177, 217–233. 10.1111/bph.14858 31479151 PMC6976785

[B56] Leo C. Hart J. Woodman O. (2011). Impairment of both nitric oxide-mediated and EDHF-type relaxation in small mesenteric arteries from rats with streptozotocin-induced diabetes. Br. J. Pharmacol. 162, 365–377. 10.1111/j.1476-5381.2010.01023.x 20840539 PMC3031058

[B57] Leo C. Joshi A. Hart J. Woodman O. (2012). Endothelium-dependent nitroxyl-mediated relaxation is resistant to superoxide anion scavenging and preserved in diabetic rat aorta. Pharmacol. Res. 66, 383–391. 10.1016/j.phrs.2012.07.010 22898326

[B58] Leung S. Vanhoutte P. (2017). Endothelium-dependent hyperpolarization: age, gender and blood pressure, do they matter?. Acta Physiol. 219, 108–123. 10.1111/apha.12628 26548576

[B59] Li C. Rand M. (1993). Effects of hydroxocobalamin and haemoglobin on no-mediated relaxations in the rat anococcygeus muscle. Clin. Exp. Pharmacol. Physiol. 20, 633–640. 10.1111/j.1440-1681.1993.tb01645.x 8261658

[B60] Lin J.-L. Sung K.-T. Su C.-H. Chou T.-H. Lo C.-I. Tsai J.-P. (2018). Cardiac structural remodeling, longitudinal systolic strain, and torsional mechanics in lean and nonlean dysglycemic Chinese adults. Circulation: Cardiovas. Imaging 11 (5), e007047. 10.1161/CIRCIMAGING.117.007047 29752393

[B61] Luiking Y. C. Engelen M. P. Deutz N. E. (2010). Regulation of nitric oxide production in health and disease. Curr. Opin. Clin. Nutr. Metab. Care 13, 97. 10.1097/MCO.0b013e328332f99d 19841582 PMC2953417

[B62] Makino A. Ohuchi K. Kamata K. (2000). Mechanisms underlying the attenuation of endothelium-dependent vasodilatation in the mesenteric arterial bed of the streptozotocin-induced diabetic rat. Br. J. Pharmacol. 130, 549–556. 10.1038/sj.bjp.0703354 10821782 PMC1572112

[B63] Marsh S. A. Dell'italia L. J. Chatham J. C. (2009). Interaction of diet and diabetes on cardiovascular function in rats. Am. J. Physiol. Heart Circ. Physiol. 296, H282–H292. 10.1152/ajpheart.00421.2008 19036853 PMC2643886

[B64] Marshall S. A. Qin C. X. Jelinic M. O’sullivan K. Deo M. Walsh J. (2020). The novel small-molecule annexin-A1 mimetic, compound 17b, elicits vasoprotective actions in streptozotocin-induced diabetic mice. Int. J. Mol. Sci. 21, 1384. 10.3390/ijms21041384 PMC7073122 32085666

[B65] Marti M. Álvarez L. Suarez S. Doctorovich F. (2017). Is azanone endogenously produced in mammals?. Boston, MA: Elsevier.

[B66] Matsumoto T. Kobayashi S. Ando M. Watanabe S. Iguchi M. Taguchi K. (2017). Impaired endothelium-derived hyperpolarization-type relaxation in superior mesenteric arteries isolated from female Otsuka Long-Evans Tokushima Fatty rats. Eur. J. Pharmacol. 807, 151–158. 10.1016/j.ejphar.2017.03.062 28433656

[B67] Matsumoto T. Kobayashi T. Kamata K. (2003). Alterations in EDHF-type relaxation and phosphodiesterase activity in mesenteric arteries from diabetic rats. Am. J. Physiol. Heart Circ. Physiol. 285, H283–H291. 10.1152/ajpheart.00954.2002 12793980

[B68] Miller T. W. Cherney M. M. Lee A. J. Francoleon N. E. Farmer P. J. King S. B. (2009). The effects of nitroxyl (HNO) on soluble guanylate cyclase activity: interactions at ferrous heme and cysteine thiols. J. Biol. Chem. 284, 21788–21796. 10.1074/jbc.M109.014282 19531488 PMC2755905

[B69] Miranda K. M. Yamada K. Espey M. G. Thomas D. D. Degraff W. Mitchell J. B. (2002). Further evidence for distinct reactive intermediates from nitroxyl and peroxynitrite: effects of buffer composition on the chemistry of Angeli's salt and synthetic peroxynitrite. Arch. Biochem. Biophys. 401, 134–144. 10.1016/S0003-9861(02)00031-0 12054463

[B70] Mokhtar S. S. Vanhoutte P. M. Leung S. W. Suppian R. Yusof M. I. Rasool A. H. (2016a). Reduced nitric oxide-mediated relaxation and endothelial nitric oxide synthase expression in the tail arteries of streptozotocin-induced diabetic rats. Eur. J. Pharmacol. 773, 78–84. 10.1016/j.ejphar.2016.01.013 26825543

[B71] Mokhtar S. S. Vanhoutte P. M. Leung S. W. Yusof M. I. Wan Sulaiman W. A. Mat Saad A. Z. (2016b). Endothelium dependent hyperpolarization-type relaxation compensates for attenuated nitric oxide-mediated responses in subcutaneous arteries of diabetic patients. Nitric Oxide. 53, 35–44. 10.1016/j.niox.2015.12.007 26768833

[B72] Moncada S. Gryglewski R. Bunting S. Vane J. (1976). An enzyme isolated from arteries transforms prostaglandin endoperoxides to an unstable substance that inhibits platelet aggregation. Nature 263, 663. 10.1038/263663a0 802670

[B73] Mulvany M. Aalkjaer C. (1990). Structure and function of small arteries. Physiol. Rev. 70, 921–961. 10.1152/physrev.1990.70.4.921 2217559

[B74] Okon E. B. Chung A. W. Rauniyar P. Padilla E. Tejerina T. Mcmanus B. M. (2005). Compromised arterial function in human type 2 diabetic patients. Diabetes 54, 2415–2423. 10.2337/diabetes.54.8.2415 16046309

[B75] Palmer R. M. Ashton D. Moncada S. (1988). Vascular endothelial cells synthesize nitric oxide from L-arginine. Nature 333, 664. 10.1038/333664a0 3131684

[B76] Pannirselvam M. Verma S. Anderson T. J. Triggle C. R. (2002). Cellular basis of endothelial dysfunction in small mesenteric arteries from spontaneously diabetic (db/db -/-) mice: role of decreased tetrahydrobiopterin bioavailability. Br. J. Pharmacol. 136, 255. 10.1038/sj.bjp.0704683 12010774 PMC1573335

[B77] Paolocci N. Biondi R. Bettini M. Lee C. I. Berlowitz C. O. Rossi R. 2001). Oxygen radical-mediated reduction in basal and agonist-evoked NO release in isolated rat heart. J. Mol. Cell. Cardiol. 33, 671–679. 10.1006/jmcc.2000.1334 11341236

[B78] Paolocci N. Keceli G. Wink D. Kass D. A. (2016). “From heaven to heart: nitroxyl (HNO) in the cardiovascular system and beyond,” in The chemistry and biology of nitroxyl (HNO) (Amsterdam, Netherlands: Elsevier Inc.), 353–387.

[B79] Park Y. Capobianco S. Gao X. Falck J. R. Dellsperger K. C. Zhang C. (2008). Role of EDHF in type 2 diabetes-induced endothelial dysfunction. Am. J. Physiol. Heart Circ. Physiol. 295, H1982–H1988. 10.1152/ajpheart.01261.2007 18790831 PMC2614585

[B80] Park Y. Yang J. Zhang H. Chen X. Zhang C. (2011). Effect of PAR2 in regulating TNF-α and NAD(P)H oxidase in coronary arterioles in type 2 diabetic mice. Basic Res. Cardiol. 106, 111–123. 10.1007/s00395-010-0129-9 20972877 PMC3143573

[B81] Pennathur S. Heinecke J. W. (2007). Oxidative stress and endothelial dysfunction in vascular disease. Curr. Diabetes Rep. 7, 257–264. 10.1007/s11892-007-0041-3 17686400

[B82] Pino R. Z. Feelisch M. (1994). Bioassay discrimination between nitric oxide (NO.) and nitroxyl (NO-) using L-cysteine. Biochem. Biophys. Res. Commun. 201, 54–62. 10.1006/bbrc.1994.1668 8198612

[B83] Podell B. K. Ackart D. F. Richardson M. A. Dilisio J. E. Pulford B. Basaraba R. J. (2017). A model of type 2 diabetes in the Guinea pig using sequential diet-induced glucose intolerance and streptozotocin treatment. Dis Model Mech. 10, 151–162. 10.1242/dmm.025593 28093504 PMC5312002

[B84] Qin C. X. Anthonisz J. Leo C. H. Kahlberg N. Velagic A. Li M. (2020). Nitric oxide resistance, induced in the myocardium by diabetes, is circumvented by the nitric oxide redox sibling, nitroxyl. Antioxidants Redox Signal. 32, 60–77. 10.1089/ars.2018.7706 31680536

[B85] Reed M. Meszaros K. Entes L. Claypool M. Pinkett J. Gadbois T. (2000). A new rat model of type 2 diabetes: the fat-fed, streptozotocin-treated rat. Metab. Clin. Exp. 49, 1390–1394. 10.1053/meta.2000.17721 11092499

[B86] Ritchie R. H. Abel E. D. (2020). Basic mechanisms of diabetic heart disease. Circ. Res. 126, 1501–1525. 10.1161/CIRCRESAHA.120.315913 32437308 PMC7251974

[B87] Ritchie R. H. Leo C. H. Qin C. Stephenson E. J. Bowden M. A. Buxton K. D. 2013). Low intrinsic exercise capacity in rats predisposes to age-dependent cardiac remodeling independent of macrovascular function. Am. J. Physiol. Heart Circ. Physiol. 304, H729–H739. 10.1152/ajpheart.00638.2012 23262135 PMC3833993

[B88] Ritchie R. Love J. E. Huynh K. Bernardo B. Henstridge D. Kiriazis H. 2012). Enhanced phosphoinositide 3-kinase(p110α) activity prevents diabetes-induced cardiomyopathy and superoxide generation in a mouse model of diabetes. Diabetologia 55, 3369–3381. 10.1007/s00125-012-2720-0 23001375

[B89] Rusche K. M. Spiering M. M. Marletta M. A. (1998). Reactions catalyzed by tetrahydrobiopterin-free nitric oxide synthase. Biochemistry (Mosc.) 37, 15503–15512. 10.1021/bi9813936 9799513

[B90] Sampath C. Rashid M. R. Sang S. Ahmedna M. (2017). Green tea epigallocatechin 3-gallate alleviates hyperglycemia and reduces advanced glycation end products via nrf2 pathway in mice with high fat diet-induced obesity. Biomed. Pharmacother. 87, 73–81. 10.1016/j.biopha.2016.12.082 28040599

[B91] Sankar P. Subhashree S. Sudharani S. (2012). Effect of Trigonella frenum-graecum seed powder on the antioxidant levels of high fat diet and low dose streptozotocin induced type II diabetic rats. Eur. Rev. Med. Pharmacol. Sci. 16 (Suppl 3), 10–17. 22957413

[B92] Schach C. Resch M. Schmid P. M. Riegger G. A. Endemann D. H. (2014). Type 2 diabetes: increased expression and contribution of IKCa channels to vasodilation in small mesenteric arteries of ZDF rats. Am. J. Physiol. Heart Circ. Physiol. 307, H1093–H1102. 10.1152/ajpheart.00240.2013 25128173

[B93] Schalkwijk C. Stehouwer C. D. 2005). Vascular complications in diabetes mellitus: the role of endothelial dysfunction. Clin. Sci. 109, 143–159. 10.1042/CS20050025 16033329

[B94] Sharma A. Bernatchez P. N. De Haan J. B. (2012). Targeting endothelial dysfunction in vascular complications associated with diabetes. Int J Vasc Med, 2012, 750126. 10.1155/2012/750126 22013533 PMC3195347

[B95] Sharma A. K. Bharti S. Ojha S. Bhatia J. Kumar N. Ray R. (2011). Up-regulation of PPARγ, heat shock protein-27 and -72 by naringin attenuates insulin resistance, β-cell dysfunction, hepatic steatosis and kidney damage in a rat model of type 2 diabetes. Br. J. Nutr. 106, 1713–1723. 10.1017/S000711451100225X 21736771

[B96] Shemyakin A. Kövamees O. Rafnsson A. Böhm F. Svenarud P. Settergren M. (2012). Arginase inhibition improves endothelial function in patients with coronary artery disease and type 2 diabetes mellitus. Circulation. 126, 2943–2950. 10.1161/CIRCULATIONAHA.112.140335 23183942

[B97] Shi Y. Ku D. D. Man R. Y. Vanhoutte P. M. (2006). Augmented endothelium-derived hyperpolarizing factor-mediated relaxations attenuate endothelial dysfunction in femoral and mesenteric, but not in carotid arteries from type I diabetic rats. J. Pharmacol. Exp. Therapeut. 318, 276–281. 10.1124/jpet.105.099739 16565165

[B98] Shimokawa H. Godo S. (2016). Diverse functions of endothelial NO synthases system: NO and EDH. J. Cardiovasc. Pharmacol. 67, 361. 10.1097/FJC.0000000000000348 26647119 PMC4863718

[B99] Sitia S. Tomasoni L. Atzeni F. Ambrosio G. Cordiano C. Catapano A. 2010). From endothelial dysfunction to atherosclerosis. Autoimmun. Rev. 9, 830–834. 10.1016/j.autrev.2010.07.016 20678595

[B100] Spagnoli L. G. Mauriello A. Sangiorgi G. Fratoni S. Bonanno E. Schwartz R. S. (2004). Extracranial thrombotically active carotid plaque as a risk factor for ischemic stroke. Jama. 292, 1845–1852. 10.1001/jama.292.15.1845 15494582

[B101] Stoll S. Nejatyjahromy Y. Woodward J. J. Ozarowski A. Marletta M. A. Britt R. D. (2010). Nitric oxide synthase stabilizes the tetrahydrobiopterin cofactor radical by controlling its protonation state. J. Am. Chem. Soc. 132, 11812–11823. 10.1021/ja105372s 20669954

[B102] Stratton I. Cull C. Adler A. Matthews D. Neil H. Holman R. (2006). Additive effects of glycaemia and blood pressure exposure on risk of complications in type 2 diabetes: a prospective observational study (UKPDS 75). Diabetologia 49, 1761–1769. 10.1007/s00125-006-0297-1 16736131

[B103] Takaki A. Morikawa K. Tsutsui M. Murayama Y. Tekes E. Yamagishi H. (2008). Crucial role of nitric oxide synthases system in endothelium-dependent hyperpolarization in mice. J. Exp. Med. 205, 2053–2063. 10.1084/jem.20080106 18695006 PMC2526200

[B104] Tantillo D. J. Fukuto J. M. Hoffman B. M. Silverman R. B. Houk K. (2000). Theoretical studies on N G-hydroxy-L-arginine and derived radicals: implications for the mechanism of nitric oxide synthase. J. Am. Chem. Soc 122, 536–537. 10.1021/ja991876c

[B105] Tare M. Kalidindi R. S. Bubb K. J. Parkington H. C. Boon W. M. Li X. (2017). Vasoactive actions of nitroxyl (HNO) are preserved in resistance arteries in diabetes. Naunyn-Schmiedeberg’s Arch. Pharmacol. 390, 397–408. 10.1007/s00210-016-1336-1 28074232

[B106] Tomioka H. Hattori Y. Fukao M. Sato A. Liu M. Sakuma I. (1999). Relaxation in different-sized rat blood vessels mediated by endothelium-derived hyperpolarizing factor: importance of processes mediating precontractions. J. Vasc. Res. 36, 311–320. 10.1159/000025659 10474044

[B107] Van Etten R. De Koning E. Verhaar M. Gaillard C. Rabelink T. (2002). Impaired NO-dependent vasodilation in patients with Type II (non-insulin-dependent) diabetes mellitus is restored by acute administration of folate. Diabetologia 45, 1004–1010. 10.1007/s00125-002-0862-1 12136399

[B108] Vanhoutte P. Shimokawa H. Feletou M. Tang E. (2017). Endothelial dysfunction and vascular disease - a 30th anniversary update. Acta Physiol. 219, 22–96. 10.1111/apha.12646 26706498

[B109] Velagic A. Qin C. Woodman O. L. Horowitz J. D. Ritchie R. H. Kemp-Harper B. K. (2020). Nitroxyl: a novel strategy to circumvent diabetes associated impairments in nitric oxide signaling. Front. Pharmacol. 11, 727. 10.3389/fphar.2020.00727 32508651 PMC7248192

[B110] Wanstall J. C. Homer K. L. Doggrell S. A. (2005). Evidence for, and importance of, cGMP-independent mechanisms with NO and NO donors on blood vessels and platelets. Curr. Vasc. Pharmacol. 3, 41–53. 10.2174/1570161052773933 15638781

[B111] Wanstall J. C. Jeffery T. K. Gambino A. Lovren F. Triggle C. R. (2001). Vascular smooth muscle relaxation mediated by nitric oxide donors: a comparison with acetylcholine, nitric oxide and nitroxyl ion. Br. J. Pharmacol. 134, 463–472. 10.1038/sj.bjp.0704269 11588100 PMC1572971

[B112] Wigg S. J. Tare M. Tonta M. A. O'brien R. C. Meredith I. T. Parkington H. C. (2001). Comparison of effects of diabetes mellitus on an EDHF-dependent and an EDHF-independent artery. Am. J. Physiol. Heart Circ. Physiol. 281, H232–H240. 10.1152/ajpheart.2001.281.1.H232 11406490

[B113] Woodman O. L. Wongsawatkul O. Sobey C. G. (2000). Contribution of nitric oxide, cyclic GMP and K+ channels to acetylcholine-induced dilatation of rat conduit and resistance arteries. Clin. Exp. Pharmacol. Physiol. 27, 34–40. 10.1046/j.1440-1681.2000.03199.x 10696526

[B114] Wynne B. M. Labazi H. Tostes R. C. Webb R. C. (2012). Aorta from angiotensin II hypertensive mice exhibit preserved nitroxyl anion mediated relaxation responses. Pharmacol. Res. 65, 41–47. 10.1016/j.phrs.2011.07.002 21767645 PMC3908541

[B115] Zardi E. M. Afeltra A. (2010). Endothelial dysfunction and vascular stiffness in systemic lupus erythematosus: are they early markers of subclinical atherosclerosis?. Autoimmun. Rev. 9, 684–686. 10.1016/j.autrev.2010.05.018 20553974

[B116] Zhang M. Lv X. Y. Li J. Xu Z. G. Chen L. (2008). The characterization of high-fat diet and multiple low-dose streptozotocin induced type 2 diabetes rat model. Exp. Diabetes Res, 2008, 704045. 10.1155/2008/704045 19132099 PMC2613511

[B117] Zou M.-H. Cohen R. A. Ullrich V. (2004). Peroxynitrite and vascular endothelial dysfunction in diabetes mellitus. Abingdon, United Kingdom: Taylor & Francis. 10.1080/10623320490482619 15370068

